# Endoglin and squamous cell carcinomas

**DOI:** 10.3389/fmed.2023.1112573

**Published:** 2023-06-16

**Authors:** Sarah K. Hakuno, Stefanus G. T. Janson, Marjolijn D. Trietsch, Manon de Graaf, Eveline de Jonge-Muller, Stijn Crobach, Tom J. Harryvan, Jurjen J. Boonstra, Winand N. M. Dinjens, Marije Slingerland, Lukas J. A. C. Hawinkels

**Affiliations:** ^1^Department of Gastroenterology-Hepatology, Leiden University Medical Center, Leiden, Netherlands; ^2^Department of Pathology, Leiden University Medical Center, Leiden, Netherlands; ^3^Department of Gynecology, Leiden University Medical Center, Leiden, Netherlands; ^4^Department of Medical Oncology, Leiden University Medical Center, Leiden, Netherlands; ^5^Department of Pathology, Erasmus MC Cancer Institute, University Medical Center, Rotterdam, Netherlands

**Keywords:** endoglin, squamous cell carcinoma, TGF-β, BMP-9, TRC105

## Abstract

Despite the fact that the role of endoglin on endothelial cells has been extensively described, its expression and biological role on (epithelial) cancer cells is still debatable. Especially its function on squamous cell carcinoma (SCC) cells is largely unknown. Therefore, we investigated SCC endoglin expression and function in three types of SCCs; head and neck (HNSCC), esophageal (ESCC) and vulvar (VSCC) cancers. Endoglin expression was evaluated in tumor specimens and 14 patient-derived cell lines. Next to being expressed on angiogenic endothelial cells, endoglin is selectively expressed by individual SCC cells in tumor nests. Patient derived HNSCC, ESCC and VSCC cell lines express varying levels of endoglin with high interpatient variation. To assess the function of endoglin in signaling of TGF-β ligands, endoglin was overexpressed or knocked out or the signaling was blocked using TRC105, an endoglin neutralizing antibody. The endoglin ligand BMP-9 induced strong phosphorylation of SMAD1 independent of expression of the type-I receptor ALK1. Interestingly, we observed that endoglin overexpression leads to strongly increased soluble endoglin levels, which in turn decreases BMP-9 signaling. On the functional level, endoglin, both in a ligand dependent and independent manner, did not influence proliferation or migration of the SCC cells. In conclusion, these data show endoglin expression on individual cells in the tumor nests in SCCs and a role for (soluble) endoglin in paracrine signaling, without directly affecting proliferation or migration in an autocrine manner.

## 1. Introduction

Cutaneous squamous cell carcinomas (cSCCs) are the second most common form of skin cancer that arise specifically from squamous epithelial cells (1). Besides the cutaneous form, SCCs can also arise inside the body. Squamous cells are characterized by their flat, sheet-like morphology and can for example be found lining the head and neck region, esophagus, and vagina (2). Cancers of the oral cavity, (naso-, oro-, and hypo-), pharynx and larynx (collectively known as head and neck cancers) combined are responsible for 444.000 cancer related deaths in 2020 worldwide (3). Most of the head and neck cancers are squamous cell carcinomas (HNSCCs) and develop from the mucosal squamous epithelium (4, 5). Esophageal carcinoma ranks sixth in mortality (544.000 deaths in 2020) worldwide (3). The two most common histological subtypes of esophageal cancer are squamous cell carcinoma and adenocarcinoma. Esophageal squamous cell carcinomas (ESCCs) are most often located in the proximal or middle part of the esophagus (6). Worldwide, ESCC is the most common subtype, whereas the esophageal adenocarcinomas are the most prevalent type in the Western world (7). Vulvar cancer is a gynecological cancer that contributed 17.000 deaths in 2020 worldwide (3), with the vast majority being squamous cell carcinomas (VSCC) (8). VSCC can be categorized as human papillomavirus (HPV)-positive and HPV-negative (9). Within the HPV-negative VSCC tumors, two main histological phenotypes have been described: the conventional phenotype and a specific subgroup (approximately 20%) that show a spindle-shaped morphology (10). This spindle cell morphology is associated with a worse prognosis than the conventional morphology and it was found that five-year survival was lower in patients with versus without spindle morphology (10).

Despite intense treatment regimens, these three types of SCCs show a high number of cancer-related deaths. Therefore, new efficient targeted therapies are needed. Endoglin (CD105), is a co-receptor for ligands of the transforming growth factor beta (TGF-β) pathway and endothelial endoglin expression is associated with metastasis and poor oncological outcome in SCC patients (11–13). The TGF-β superfamily consists of multiple cytokines, including the TGF-βs and bone morphogenic proteins (BMPs) (14). During canonical TGF-β signaling, TGF-β binds to the TGF-β type II receptor, which phosphorylates the serine/threonine kinase domain of a TGF-β type I receptor, also known as the activin receptor-like kinase (ALK)-5. The activated type I receptor induces phosphorylation of SMAD2/3. Binding to SMAD4 and translocation to the nucleus leads to gene transcription of multiple target genes (14, 15). However, when endoglin is expressed, for example on proliferating endothelial cells where it plays a part in angiogenesis (16), the type I TGF-β receptor ALK1 is recruited, leading to the phosphorylation of SMAD1/5/9, and transcription of different target genes (17, 18). Both TGF-β and BMP-9 can bind to endoglin and induce phosphorylation of SMAD1/5/9 (19).

In multiple types of cancer, it has been shown that a high endoglin expression on tumor vessels is correlated with a worse clinical outcome (20, 21). Consequently, an endoglin neutralizing antibody has been developed, TRC105. TRC105 is an IgG1 monoclonal endoglin-neutralizing antibody that prevents BMP-9 ligand binding and thereby inhibits tumor angiogenesis (22, 23). Even though endoglin is predominantly expressed on endothelial cells, endoglin expression has also been observed in other cell types, including cancer-associated fibroblasts (CAFs) (24–26), regulatory T cells (27), and epithelial tumor cells (28). On CAFs, endoglin has been shown to promote tumor progression and metastasis (24, 25). However, the role of endoglin expression on epithelial cancer cells is more controversial. Epithelial endoglin expression has been described to have a tumor-suppressive function in lung cancer, breast cancer, prostate cancer, and ESCC (29–32). On the contrary, other studies have described it to have a pro tumorigenic role in renal cell carcinoma and also non-epithelial cancers such as, melanoma, leukemia, and Ewing sarcoma (33–35). Consequently, the exact role of endoglin on epithelial tumor cells, and especially SCC, remains not understood. Therefore, we set out to investigate endoglin expression of squamous epithelial cancer cells in patient samples from ESCC, VSCC, and HNSCC and studied the role of endoglin in influencing TGF-β/BMP-9 signaling *in vitro*.

## 2. Materials and methods

### 2.1. Patient samples

Formalin fixed paraffin-embedded (FFPE) tissue samples were obtained from the Department of Surgery, Leiden University Medical Center (LUMC, Leiden, the Netherlands). Samples were tumor tissue obtained after surgery for HNSCC (*n* = 5), ESCC (*n* = 11), or VSCC (*n* = 7). Samples were used in an anonymized manner and according to the guidelines of the Medical Ethical Committee of the LUMC, and conducted in accordance to the Declaration of Helsinki and the Code of Conduct for responsible use of Human Tissue and Medical Research as drawn up by the Federation of Dutch Medical Scientific Societies in 2011. This Code permits the further use of coded residual (historical) tissue and data from the diagnostic process for scientific purposes.

### 2.2. Immunohistochemistry

Immunohistochemistry was performed as described before (23). In short, FFPE sections (4 μm) were deparaffinized, blocked in 0.3% hydrogen peroxide (H_2_O_2_) in methanol for 20 min, and rehydrated. Antigen retrieval was performed by boiling the sections in a 0.01 M citrate solution (pH 6.0) for 10 min. After being washed with phosphate-buffered saline (PBS), the sections were incubated with polyclonal goat anti-human endoglin (1:400—BAF1097, R&D systems, MN, United States) in PBS/1% bovine serum albumin (BSA), and left overnight at room temperature. The next day, the slides were washed and then incubated with a biotinylated polyclonal rabbit anti goat secondary antibody (Dako, CA, United States) for 30 min, washed and incubated with Vectastain complex (Vector Laboratories, CA, United States). The color was developed using a 3,3’diaminobenzidine (DAB) + substrate chromogen system (Dako, CA, United States), following the manufacturer instructions. Nuclear staining was performed using hematoxylin (Merck, Darmstadt, Germany). Slides were dehydrated and mounted using entellan (Merck, Darmstadt, Germany).

### 2.3. Imaging mass cytometry

Imaging mass cytometry with the Hyperion mass cytometry system was performed as described before (36). In short, antibodies were conjugated to purified lanthanide metals (Fluidigm, CA, United States; Table 1) using the MaxPar X8 antibody labeling kit and protocol (Fluidigm). 4 μm FFPE sections were deparaffinized, rehydrated and antigen retrieval (high pH—pH9 Thermo Fisher Scientific) was performed by boiling the sections in the microwave. Sections were incubated for 30 min with Superblock solution, after which, excess Superblock was tapped off. Sections were incubated with antibodies according to Table 1. Following, sections were incubated with Intercalator-Ir (125 μM, Fluidigm) for 5 min. The slides were then dried under an airflow and stored at room temperature until ablation. Prior to acquisition, the Hyperion mass cytometry system (Fluidigm) was autotuned using a three-element tuning slide (Fluidigm) according to the tuning protocol provided by the Hyperion imaging system user guide (Fluidigm). Regions of interest were selected based on hematoxylin and eosin stains and pan-cytokeratin IHC, after which areas of 1,000 × 1,000 μm were ablated and acquired at 200 Hz. Data was exported as MCD files and visualized using the Fluidigm MCD™ viewer.

**Table 1:** Six marker FFPE panel for imaging mass spectrometry (Hyperion).

Target	Clone	Metal	Time	Temperature	Dilution
Endoglin + 2nd AB (biotin)	BAF1097	147 Sm	indirect O/N + 1 h	4° C + rT	1:50 + 1:100
Pan-cytokeratin	AE1/AE3	148 Nd	5 h	rT	1:100
p53	7F5	163 Dy	O/N	4° C	1:100
CD45	D9M8I	145 Nd	O/N	4° C	1:50
CD68	D4B9C	157 Gd	O/N	4° C	1:100
Vimentin	D21H3	143 Nd	5 h	rT	1:200

AB, antibody; O/N, overnight; and rT, room temperature.

### 2.4. Patient-derived cell lines and cell culture

For HNSCC, oral squamous carcinoma (OSC-19) cells, a human SCC cell line derived from tongue tumor and FaDu cells (hypopharyngeal SCC) were used (37). Ten patient derived ESCC cell lines (TE01, TE02, TE04-TE06, TE08, TE10, TE11, TE14, and TE15) were obtained from the Erasmus MC Cancer Institute, University Medical Center (Rotterdam, the Netherlands) (38). Three patient derived VSCC cell lines were obtained from the LUMC (Leiden, the Netherlands) (39). From one VSCC patient sample, conventional (VC415-C) and spindle-shaped (VC415-S) VSCC cells were isolated, allowing a direct comparison (images of morphologies can be found in Supplementary Figure 1). OSC-19 and ESCC cells were cultured in Roswell Park Memorial Institute (RPMI) 1640 medium supplemented with 25 mM HEPES (Thermo Fisher Scientific, MA, United States), 10% fetal calf serum (FCS—HyClone Laboratories, UT, United States), 100 IU/mL penicillin, and 100 μg/mL streptomycin (both from Thermo Fisher Scientific, MA, United States). VSCC cells were cultured in Dulbecco’s modified Eagle’s medium (DMEM)/F12 medium (Thermo Fisher Scientific, MA, United States) supplemented with 10 mM HEPES (Thermo Fisher Scientific, MA, United States), 10% FCS, 100 IU/mL penicillin, and 100 μg/mL streptomycin. FaDu cells were cultured in DMEM (low glucose—Thermo Fisher Scientific, MA, United States) supplemented with 10% FCS and 100 IU/mL penicillin and 100 μg/mL streptomycin. The cells were cultured at 37°C with 5% CO_2_ and were tested monthly for mycoplasma contamination.

### 2.5. Real-time quantitative PCR

Total RNA was isolated using the NucleoSpin RNA isolation kit (Macherey-Nagel, Dueren, Germany) according to the manufacturer instructions. cDNA was synthesized with the RevertAid First strand cDNA synthesis kit (Thermo Fisher Scientific, MA, United States) using 0.5–1.0 μg as RNA input. qPCR was performed with SYBR Green Master mix (Bio-Rad, CA, United States) using the iCycler Thermal Cycler and iQ5 Multicolour RT-PCR Detection System (Bio-Rad, CA, United States). Target genes were amplified using specific primers, described in Supplementary Table 1. Target gene expression levels were normalized to β-actin expression. Ct values of >35 were considered as non-detectable.

### 2.6. Signaling assays and western blot

Squamous cell carcinoma cells were seeded in six-well plates (Corning Incorporated, ME, United States) and upon 70–80% confluency, they were serum-starved for 7 h in serum-free RPMI 1640 medium. After starvation, the cells were stimulated for 1 h with either 50 ng/mL BMP-6 (Peprotech, NJ, United States), 1–2 ng/mL BMP-9 (R&D systems, MN, United States), or 5 ng/mL TGF-β1 (Peprotech, NJ, United States). For inhibition studies, 40 μg/mL human IgG (BioXCell, NH, United States) or 40 μg/mL TRC105 (TRACON Pharmaceuticals, CA, United States) was added. The IgG control and the TRC105 antibodies were added 30 min before stimulation with BMP-9.

After stimulation, the cells were lysed in RIPA buffer [250 mM NaCl, 2% NP-40 substitute, 0.5% deoxycholate, 0.1% SDS, 50 mM Tris (pH 8.0), and 2.5 mM EDTA] and protein content was determined via DC protein assay according to the manufacturer instructions (Bio-Rad, CA, United States). Western blot analysis was performed as described before (23). Membranes were incubated overnight with primary antibodies against endoglin (BAF1097, R&D systems), phosphorylated SMAD1/5/9 (clone D5B10, #13820, Cell Signaling Technology, MA, United States), and phosphorylated SMAD2 (clone 138D4, #3108, Cell Signaling Technology, MA, United States) or an in house antibody for pSMAD2 (40). An antibody against β-actin (#sc-47778, Santa Cruz Biotechnology, TX, United States) was used as a loading control.

### 2.7. Enzyme-linked immunosorbent assay

The levels of membrane bound endoglin were measured in cell lysates, while the levels of soluble endoglin were measured in the conditioned medium of the cells by ELISA as described before (41). In short, cells were lysed in RIPA buffer and protein content was determined via DC protein assay. ELISA was performed with a human endoglin DuoSet with a substrate reagent pack according to the manufacturers’ instructions (both R&D systems, MN, United States). Endoglin levels were corrected for total protein content of the cell lysates and expressed in pg./mg for lysates or pg./mL for conditioned medium.

### 2.8. Endoglin knockout, knockdown, and overexpression

For all lentiviral constructs, third-generation packaging vectors and HEK293T cells were used for the generation of lentiviral particles (42) in a biosafety level 2 laboratory (BSL-2). To generate an endoglin knockout in the ESCC cell line TE01, a sgRNA, 5′-caccgCACGT GGACAGCATGGACCG-3′ (lowercase nucleotides are compatible with the restriction site) targeting exon 1 was cloned into *BsmBI*-digested plentiCRISPRv2-puromycin [Addgene: 98290 (43);]. After transduction, puromycin-resistant TE01 cells that lost endoglin expression were FACS-sorted and subsequently expanded to acquire a polyclonal TE01 endoglin knockout line. Cells were selected and cultured with 2 μg/mL of puromycin (Sigma-Aldrich, MO, United States). The VC415-S cell line was used to generate an endoglin knockdown by use of endoglin targeting short hairpin RNAs (shRNA). Six different shRNA constructs were applied: SHC002 (non-targeting control), TRCN0000083140, and TRCN0000083141 (Sigma Mission shRNA library). Once the cells reached 80% confluency, lentiviral transduction was initiated. After a 48 h period of infection, stable clones were selected by puromycin selection (1.5 μg/mL, Sigma-Aldrich). During culturing, the cells were kept under continuous puromycin pressure. Endoglin overexpression in the ESCC cell lines TE10 and TE11 was accomplished by lentiviral transduction with empty vector and CMV.ENDOGLIN.IRES.GFP lentiviruses as described before (24).

### 2.9. MTS proliferation assay

Squamous cell carcinoma cells (4,000–5,500 cells per well) were seeded in 96-well plates (Corning Incorporated, ME, United States) in triplicate. After 16 h, medium was replaced with 100 μL medium with 10% FCS or FCS-free medium supplemented with either 1–2 ng/mL BMP-9 (R&D systems, MN, United States) or 5 ng/mL TGF-β1 (Peprotech, NJ, United States). At indicated time points, 20 μL MTS substrate (Promega, WI, United States) was added to each well and absorbance was measured at 490 nm with the Cytation 5 (Biotek, CA, United States).

### 2.10. Wound healing assay

Squamous cell carcinoma cells were seeded in 48-well plate (Corning Incorporated, ME, United States) in triplicate for each condition (300.000 cells/well). After a confluent monolayer was formed, a scratch was made with a 200 μL pipette tip. After placing the scratch, plates were washed, to remove floating cells. Following, either medium with 10% FCS was added, or FCS-free medium supplemented with either 1–2 ng/mL BMP-9 (R&D systems), 5 ng/mL TGF-β1 (Peprotech), 40 μg/mL hIgG (Bio × Cell), or 40 μg/mL TRC105 (TRACON Pharmaceuticals). Images were obtained at different time points, and the open area of the open surface was quantified with ImageJ software (National Institutes of Health, MD, United States).

### 2.11. Statistical analysis

Data are presented as means ± SD. Unpaired *t* tests were used to compare two groups. One-way ANOVA was used to compare multiple groups. All analyses were performed using GraphPad Prism software (CA, United States). Values of *p* ≤ 0.05 were considered statistically significant.

## 3. Results

### 3.1. Endoglin is expressed by SCC cells in patient material

**Figure 1:**
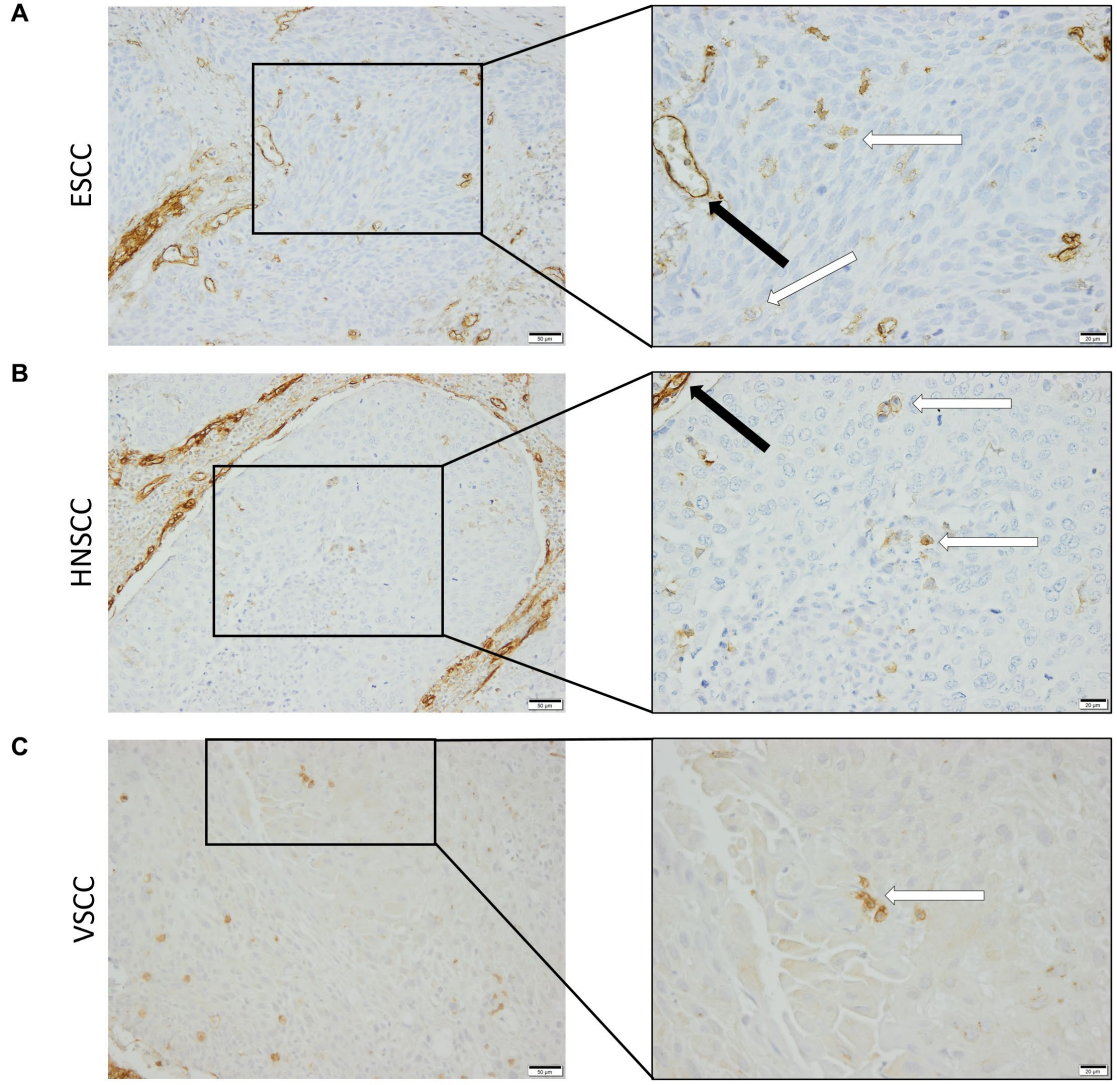
Analysis of squamous cell carcinoma (SCC) primary tumors via immunohistochemistry, all tissues were stained for endoglin expression (brown). The black arrows indicate endothelial endoglin expression. The white arrows indicate epithelial endoglin expression. **(A)** Representative images of ESCC (*n* = 9). **(B)** Representative images of HNSCC (*n* = 5). **(C)** Representative images of VSCC (*n* = 7). Images taken at 100x (left) and 200x (right).

Despite the fact that endoglin was originally identified as a marker for newly formed endothelial cells, more recent works show selective expression on a variety of cells in various (pathologic) conditions. Endoglin expression on epithelial tumor cells, however, has been a subject of debate for some years, with a limited number of published studies. Therefore, we wanted to analyze endoglin expression in three squamous cell carcinoma types: HNSCC, ESCC, and VSCC. Firstly, we analyzed tumor cell specific endoglin expression in these tumors using patient samples. Immunohistochemical analysis was performed on five HNSCCs, 11 ESCCs, and seven VSCCs. Specific expression of endoglin on epithelial tumor cells was evaluated. Immunohistochemical analysis showed high endoglin expression on newly formed blood vessels, as previously reported, together with tumor type dependent staining of stromal cells in all SSCs evaluated. When epithelial endoglin expression was assessed, we found endoglin expression in nine of the 11 (81.8%) ESCCs, five of the 5 (100%) HNSCCs, and seven of the 7 (100%) VSCCs. We observed that individual cells in the tumor islands show high endoglin expression, while the majority of the SCC cells do not (Figure 1). To explore the endoglin expression by VSCC cells with spindle morphology, we performed a triple immunofluorescent staining for endoglin, keratin, and p53 on vulvar cancer tissue, where we observed high and enriched expression of endoglin by the spindle shaped VSCC cells (Supplementary Figure 2). These data show that endoglin is expressed by particular squamous epithelial cancer cells within the tumor nests.

**Figure 2:**
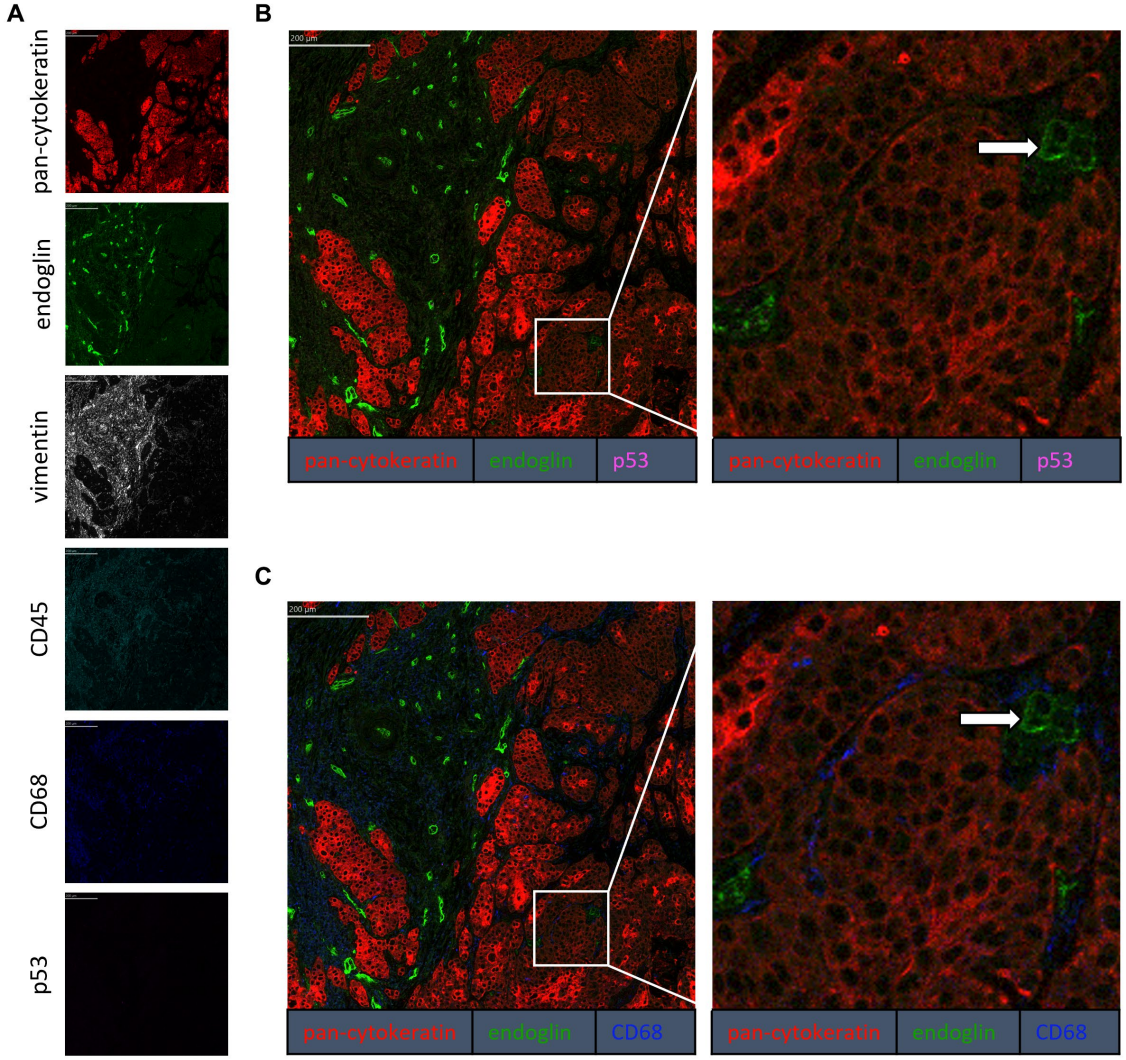
Analysis of HNSCC via imaging mass spectrometry (Hyperion) with a six marker panels. **(A)** Six images, each depicting the expression of the corresponding marker. **(B)** A merged image combining pan-cytokeratin, endoglin, and p53 expression. The white arrow indicates cells that co-express pan-cytokeratin and endoglin, which are negative for p53. **(C)** A merged image combining pan-cytokeratin, endoglin, and CD68 expression. The white arrow indicates cells that co-express pan-cytokeratin and endoglin, which are negative for CD68.

**Figure 3:**
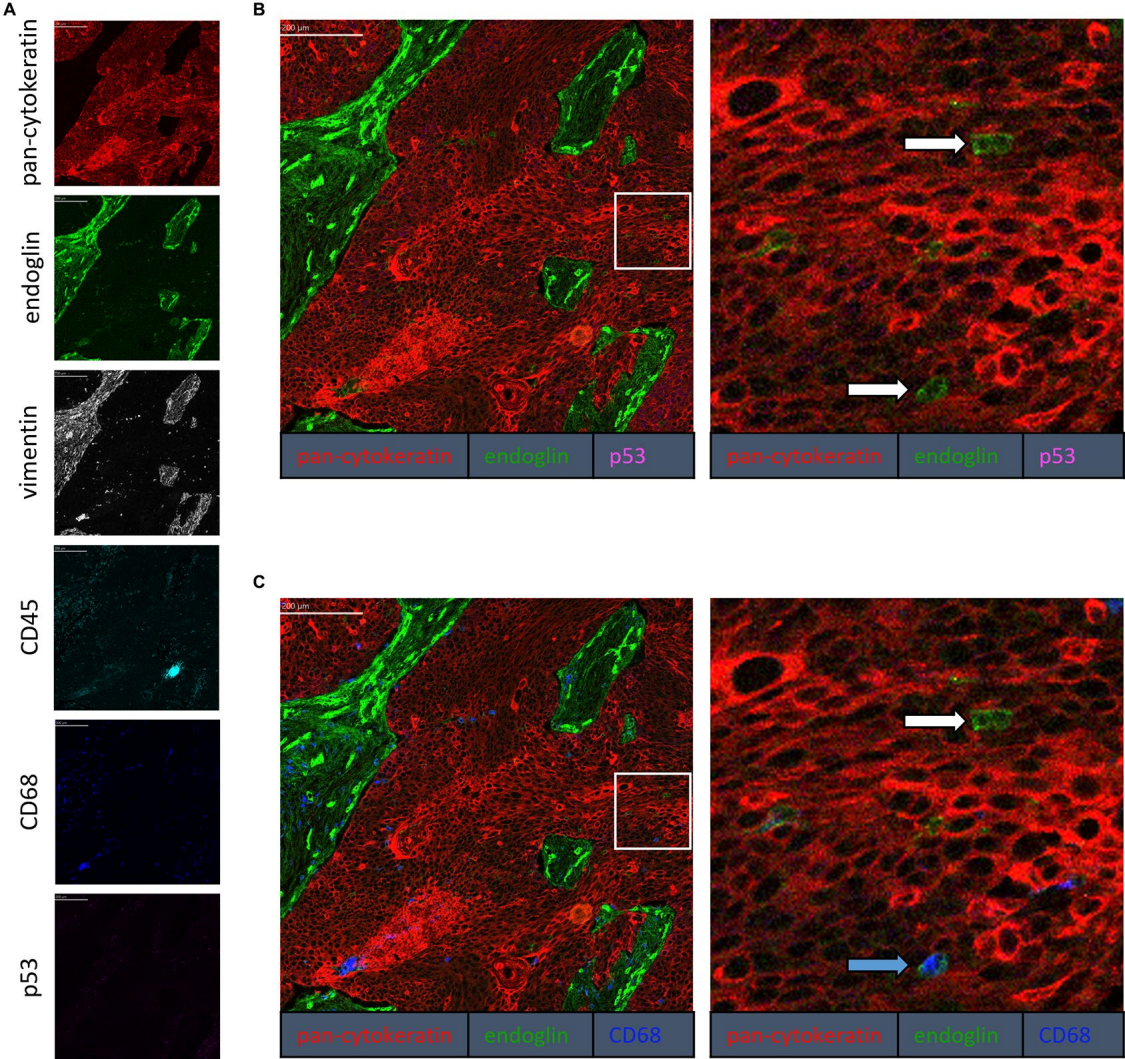
Analysis of ESCC via imaging mass spectrometry (Hyperion) with a six marker panels. **(A)** Six images, each depicting the expression of the corresponding marker. **(B)** A merged image combining pan-cytokeratin, endoglin, and p53 expression. The white arrows indicate cells that co-express pan-cytokeratin and endoglin, which are negative for p53. **(C)** A merged image combining pan-cytokeratin, endoglin, and CD68 expression. The white arrow indicates cells that co-express pan-cytokeratin and endoglin, which are negative for CD68. The blue arrow indicates cells that co-express pan-cytokeratin, endoglin, and CD68.

**Figure 4:**
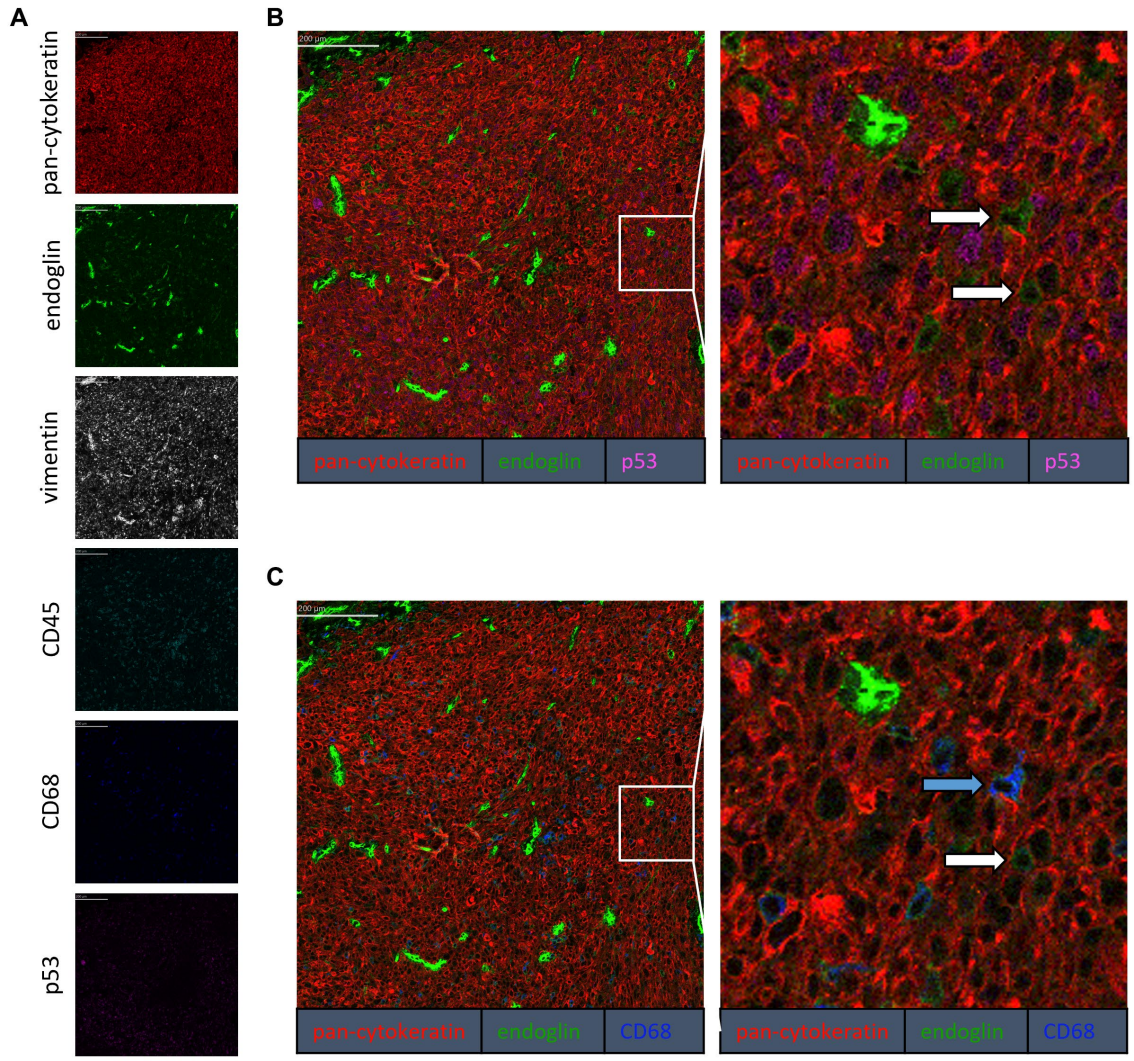
Analysis of VSCC via imaging mass spectrometry (Hyperion) with a six marker panel. **(A)** Six images, each depicting the expression of the corresponding marker. **(B)** A merged image combining pan-cytokeratin, endoglin, and p53 expression. The white arrows indicate cells that co-express pan-cytokeratin and endoglin. **(C)** A merged image combining pan-cytokeratin, endoglin, and CD68 expression. The white arrow indicates cells that co-express pan-cytokeratin and endoglin, which are negative for CD68. The blue arrow indicates cells that co-express pan-cytokeratin, endoglin, and CD68.

To confirm that the cells in the tumor expressing endoglin are indeed epithelial cancer cells and not macrophages, which were also shown to express endoglin (44), we performed imaging mass spectrometry for each tumor type (Hyperion) with a six marker panel (pan-cytokeratin, endoglin, vimentin, CD45, CD68, and p53). In the HNSCC sample, cells co-expressing endoglin and pan-cytokeratin were observed (Figure 2). In this sample, no cells co-expressing CD68 (macrophage marker) and endoglin were observed. However, in ESCC, cells that both expressed endoglin and pan-cytokeratin were detected, as well as cells that co-expressed endoglin and CD68. This indicates that individual tumor cells express endoglin, alongside with endoglin expression by macrophages (Figure 3). In conventional VSCC, we found once again both tumor cells and macrophages expressing endoglin (Figure 4). In addition, the majority of epithelial tumor cells were positive for p53 expression, indicating presence of mutant p53.

### 3.2. Endoglin expression varies between SCC cell lines

**Figure 5:**
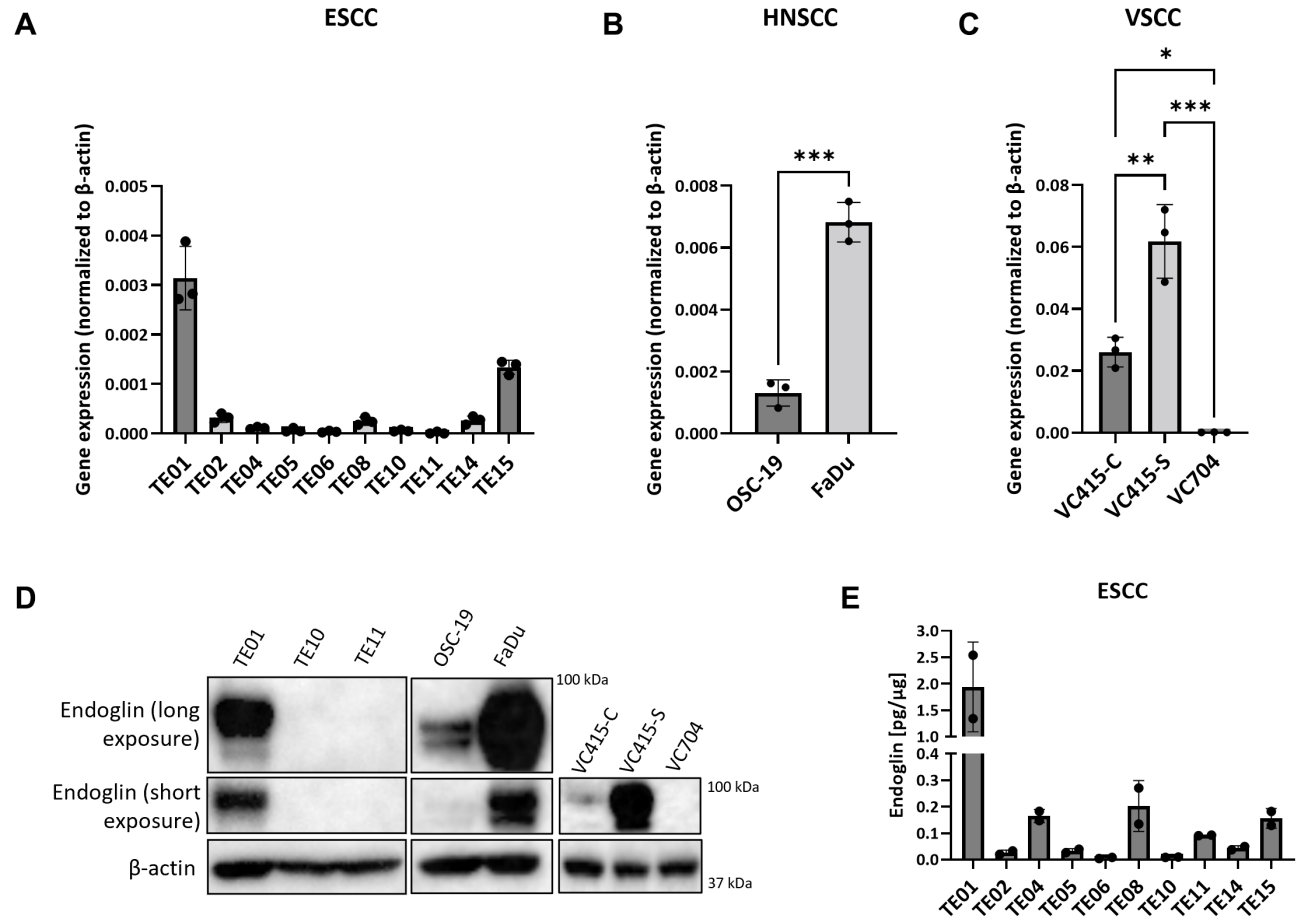
The expression of endoglin by SCC cell lines. Expression by 10 ESCC cell lines, where endoglin expression by TE01 ( *p* < 0.0001) and TE15 (*p* < 0.003) significantly differ from all other cell lines **(A)**, OSC-19 and FaDu show significantly different endoglin expression (*p* = 0.0002) **(B)**, as is also detected in the three VSCC cell lines (*p = 0.0123, **p = 0.0025, ***p = 0.0001) with different morphologies (conventional—VC415-C and VC704; spindle—VC415-S) **(C)**. Endoglin protein levels were determined via western blot **(D)** and ELISA. Endoglin protein expression by TE01 significantly differs from all other cell lines—(*p* ≤ 0.0018) **(E)**. Image for western blot analysis is a representative image of *n* = 2–3 independent experiments.

Given that in patient material, individual SCC cells are endoglin positive, but the majority is endoglin negative, we wanted to evaluate how this is reflected in SCC cell lines, to further investigate how endoglin can affect the biological behavior of SSC cells. Therefore, endoglin expression was investigated using qPCR, western blot, and ELISA analysis. Endoglin mRNA was expressed at varying levels in 10 ESCC cell lines (obtained from 10 patients). Out of these 10 cell lines, TE01 and TE15 show relatively high endoglin mRNA expression (Figure 5A). However, most ESCC lines show low or no detectable expression of endoglin. The HNSCC cell line OSC-19 shows moderate endoglin mRNA expression, whereas the FaDu cell line shows high endoglin expression (Figure 5B). When analyzing the VSCC cell lines, the cells displaying spindle morphology (VC415-S; Figure 5C) show much higher endoglin expression compared to their conventional counterpart (VC415-C; Figure 5C) and to conventional VC704, a VSCC cell line derived from another patient (Figure 5C). To further explore if these mRNA levels are also reflected in protein expression, western blot analysis was performed. The high endoglin mRNA expression in TE01 was reflected at the protein level, while TE10 and TE11show hardly any endoglin expression (Figure 5D, left panel) as expected from the mRNA levels. The HNSCC OSC-19 cells show low but detectable endoglin expression (Figure 5D, left panel) and FaDu cells show high endoglin protein expression. Finally, the conventional VSCC lines VC415-C and VC704 show low or no detectable endoglin protein expression, while the spindle cells VC415-S show very high endoglin expression (Figure 5D, right panel). When the ESCC cell lines were analyzed for endoglin protein content by ELISA, both TE01 and TE15 showed high levels of endoglin, with the other cell lines showing variable (low) amounts of endoglin (Figure 5E). These data indicate varying protein expression of endoglin on ESCC and HNSCC cells with high interpatient variation. For VSCCs, expression by spindle phenotype VSCC cells seems to be strongly increased compared to conventional VSCC cells.

### 3.3. Endoglin and SCC ALK expression

**Figure 6:**
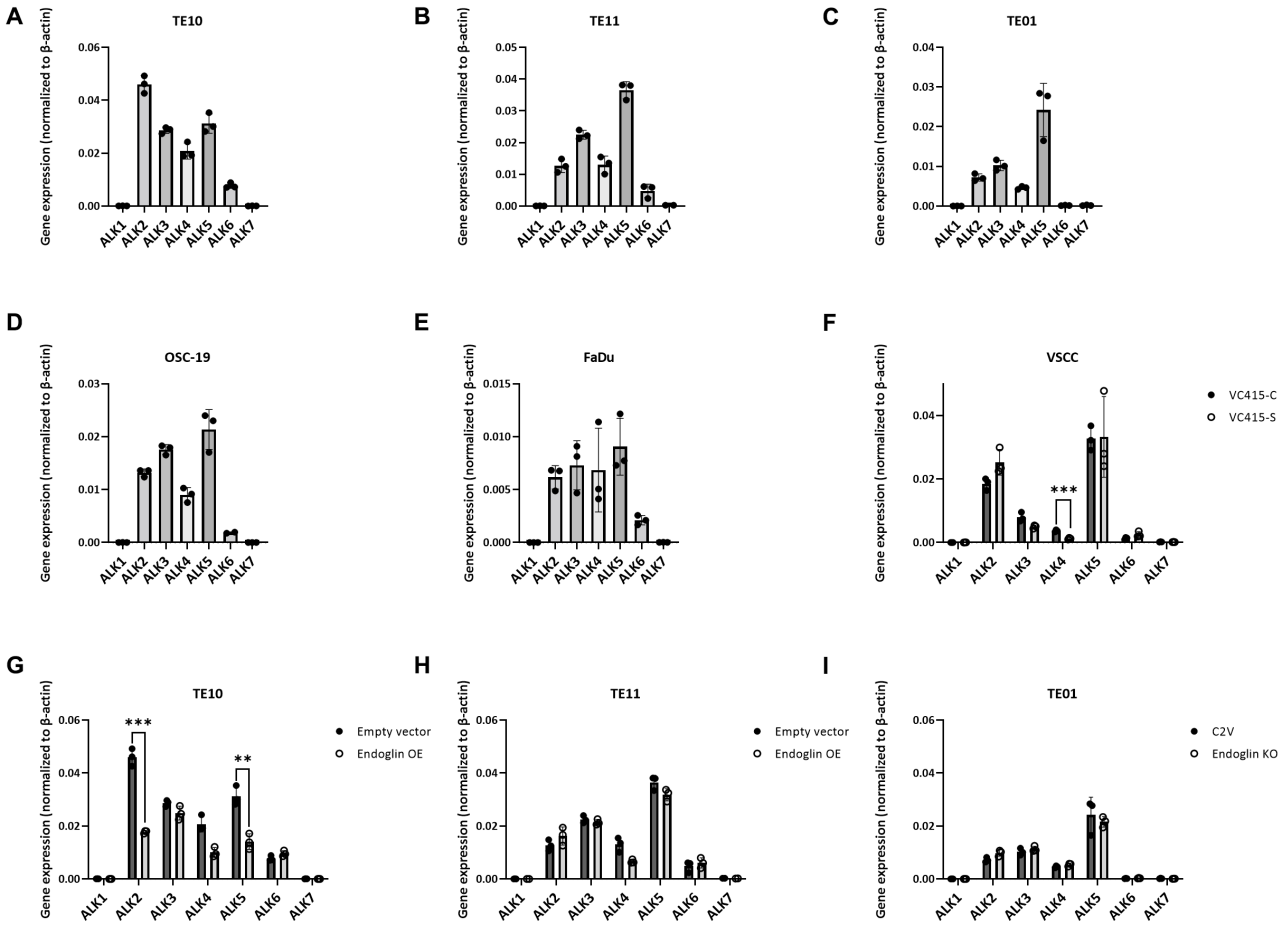
Analysis of ALK expression by SCC cell lines, determined via qPCR. Gene expression for ALK1—ALK7 by TE10—endoglin low **(A)**, TE11—endoglin low **(B)**, TE01—endoglin high **(C)**, OSC-19—endoglin positive **(D)**, FaDu–endoglin high **(E)**, VC415-C—endoglin low, and VC415-S—endoglin high; ***p = 0.000581 **(F)**. The effect of endoglin overexpression (OE) on ALK expression was analyzed for TE10; ***p=0.000126; **p=0.003291 **(G)** and TE11 **(H)**, as well as the effect of endoglin knockout (KO) in TE01 **(I)**.

Having established that SCC cells express endoglin at variable levels, we further investigated how endoglin can affect BMP and TGF-β signaling in SCC cells. In endothelial cells, endoglin is a key regulator for signaling of ligands of the TGF-β family, by shifting the pathway toward the SMAD1 pathway via an interaction with the type I receptor ALK1, instead of ALK5. Therefore, the expression of all known ALKs was evaluated on the SCC cell lines via qPCR. The low endoglin expressing ESCC cell line TE10 shows expression of ALK2, −3, −4, −5, and − 6 while ALK1 and − 7 were not detectable (Figure 6A). Similar expression was observed for the TE11 cell line (Figure 6B) and the high endoglin expressing line TE01, where in contrast, ALK6 was not expressed (Figure 6C). Similar to TE10 and TE11, the HNSCC cells OSC-19 and FaDu express ALK2, −3, −4, −5, and − 6 but do not express ALK1 or ALK7 (Figures 6D,E). For the VSCC cells, similar observations were made: ALK1, −6 and −7 are not expressed, while ALK2, −3 and − 5 are detectable and their expression does not differ significantly between the spindle (high endoglin expression) and the conventional cells (low endoglin expression) from the same patient (Figure 6F). In contrast, ALK4 is also expressed, but significantly lower in the spindle cells. Taking these data together, this suggests that, surprisingly, ALK1 is not expressed, while all SCCs show expression of ALK2, −3, and − 5.

### 3.4. Endoglin and SCC TGF-β signaling

**Figure 7:**
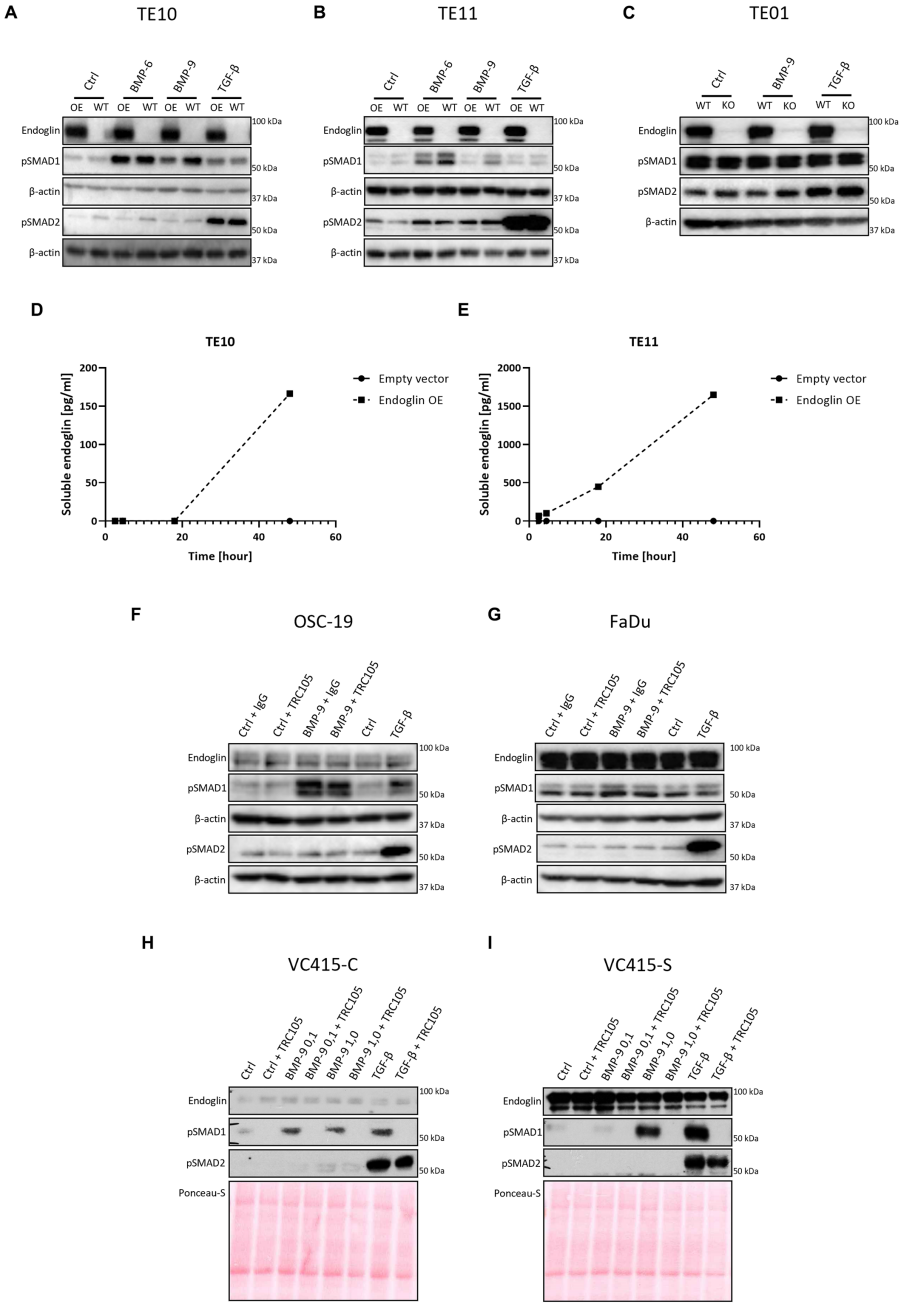
SCC cells were stimulated with either BMP-6 (TE10 and TE11 only), BMP-9 or TGF-β and the level of phosphorylated SMAD1 and SMAD2 (pSMAD1 and pSMAD2) was determined via western blot **(A–C)**. The amount of soluble endoglin in the medium of TE10 and TE11 was determined via ELISA **(D,E)**. Stimulation of OSC-19 **(F)**, FaDu **(G)**, VC415-C **(H)**, and VC415-S **(I)** with BMP-9/TGF-β/TRC105 was performed, and the levels of pSMAD1 and pSMAD2 were determined via western blot. Western blot images are representative of *n* = 2–3 per experiment.

Next, we evaluated activity of the endoglin pathway and endoglin dependent regulation of the BMP/TGF-β signaling pathway in SCC cells. Therefore, we used two approaches: Endoglin overexpression (OE) in endoglin low ESCC cells and CRISPR/Cas9 mediated endoglin knockout (KO) in high endoglin expressing ESCC cells. Absence of endoglin expression was confirmed via flow cytometry (Supplementary Figure 3). The second approach was pharmacological targeting of endoglin using the endoglin neutralizing antibody TRC105. Introduction of the endoglin OE construct results in strong endoglin expression in the low endoglin expressing lines TE10 and TE11, while the CRISPR mediated KO of endoglin in TE01 resulted in no detectable endoglin expression (Figures 7A–C). Next, to exclude any potential effect not directly related to endoglin expression, we investigated if endoglin OE or KO affects expression of the type-I receptors ALK1-7. In TE11, OE of endoglin did not affect the expression of any of the ALK receptors, while in TE10 a decrease in ALK2 and ALK5 mRNA was observed (Figures 6G,H). Endoglin KO in TE01 did not affect mRNA expression of any of the ALKs (Figure 6I).

Next, we set out to investigate the role of SCC epithelial endoglin expression on activity of the TGF-β pathway. ESCC cells were stimulated with the endoglin ligands BMP-9 and TGF-β, which can both signal via endoglin, while TGF-β can also directly induce signaling in an endoglin independent manner through TβRII/ALK5 interaction. This leads to downstream phosphorylation of SMAD1 and SMAD2 signaling molecules, respectively. BMP-6 stimulation, which acts independent of endoglin via a BMPRII/ALK3 interaction, was taken along as a control.

Low endoglin expressing ESCC cells TE10 and TE11 showed robust SMAD1 phosphorylation upon BMP-6 and BMP-9 stimulation (Figures 7A,B). Surprisingly, OE of endoglin in these cells led to a decrease in BMP-9 induced SMAD1 phosphorylation, while BMP-6 induced SMAD1 phosphorylation was not affected in TE10 and slightly reduced in TE11. Total levels of SMAD1 and SMAD2 were unaffected upon stimulation (Supplementary Figure 4). TGF-β induced SMAD2 phosphorylation was strong and not affected by endoglin OE. To further study this, we stimulated the high endoglin expressing TE01 cells and checked downstream signaling. Interestingly, these cells show high basal SMAD1 and SMAD2 phosphorylation (Figure 7C). Stimulation with BMP-9 did not further increase pSMAD1, neither did KO of endoglin affect the pSMAD1 levels. TGF-β induced strong SMAD2 phosphorylation, independent of endoglin expression. Taken together, these data indicate that endoglin KO does not directly influence SMAD1 dependent signaling, while OE of endoglin even seems to reduce BMP-9 induced pSMAD1. A potential explanation for the decrease of BMP-9 induced pSMAD1 could be increased endoglin shedding. Therefore, we measured soluble endoglin levels in the medium of TE10 and TE11. OE of endoglin indeed leads to a strongly increased amount of soluble endoglin in time, both in TE10 and TE11, when compared to the empty vector control (Figures 7D,E).

To further explore the role of endoglin in BMP-9 induced signaling, we used an endoglin neutralizing antibody that specifically inhibits BMP-9 binding to endoglin (19). OSC-19 and FaDu HNSCC cells were stimulated with BMP-9 in the presence of an IgG control or TRC105. BMP-9 induced SMAD1 phosphorylation, which could not be inhibited by TRC105 (Figures 7F,G). The low endoglin expressing VC415-C VSCC cells showed weak SMAD1 phosphorylation, which was inhibited by TRC105 (Figure 7H). High endoglin expressing spindle VSCC cells VC415-S showed strong SMAD1 phosphorylation, which could be completely inhibited by TRC105, indicating that BMP-9 induced SMAD1 phosphorylation is endoglin dependent in these cells (Figure 7I).

### 3.5. Endoglin expression does not affect SCC cell migration or proliferation *in vitro*

Given the varying expression of endoglin on SCC cells and the varying degree to which it is required for signaling, together with reported data that endoglin can affect the migratory capacity of endothelial- and tumor cells via interactions with integrins (45), we further investigated the functional effect of endoglin on SCC cell proliferation and migration.

**Figure 8:**
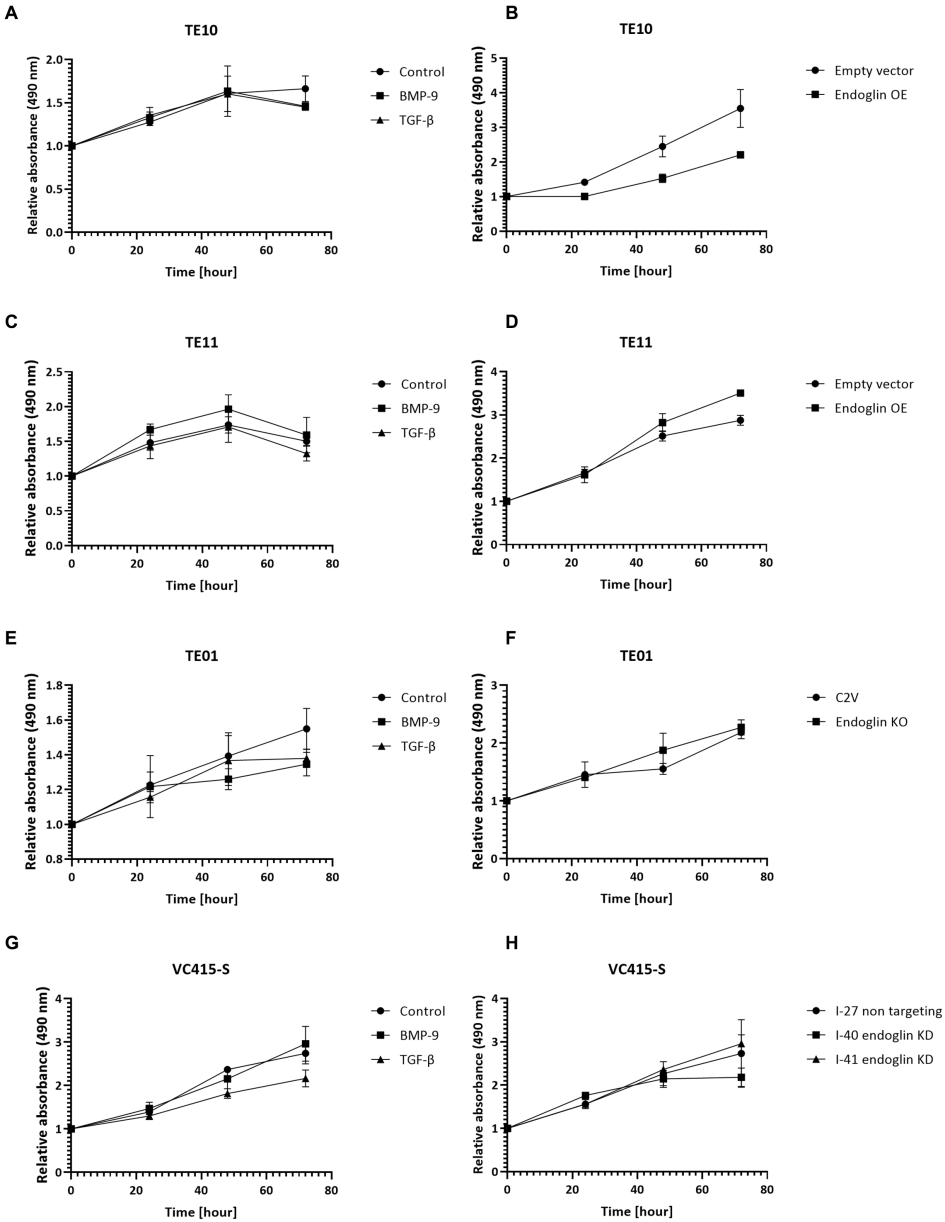
SCC cell lines were stimulated with BMP-9 or TGF-β and the proliferation of TE10 **(A)**, TE11 **(C)**, TE01 **(E)**, and VC415-S **(G)** cells was measured via a MTS assay. To assess the effects of endoglin on cell proliferation, MTS assays were performed on endoglin overexpressing (OE) TE10 **(B)** and TE11 **(D)** cells. The effects of endoglin knockout (KO) in TE01 **(F)** and endoglin knockdown (KD) in VC415-S **(H)** were also assessed via MTS. *n* = 2–3 for each experiment.

The proliferation of low endoglin expressing TE10 and TE11 ESCC cells was investigated under control conditions and after stimulation with TGF-β and BMP-9. Neither one of the ligands affected proliferation (Figures 8A,C). In contrast, OE of endoglin in TE10 led to slightly decreased proliferation of TE10 cells in a ligand independent manner, while in TE11 no significant changes were observed (Figures 8B,D). In the high endoglin expressing TE01 ESCC cells, TGF-β or BMP-9 did not influence proliferation, neither did KO of endoglin effect the proliferative capacities of these cells (Figures 8E,F). Similar observations were made for the high endoglin expressing VSCC cells VC415-S; no effect of TGF-β or BMP-9 stimulation on cellular proliferation as well as no effects of shRNA mediated endoglin knockdown (KD) in these cells compared to the non-targeting control (Figures 8G,H).

**Figure 9:**
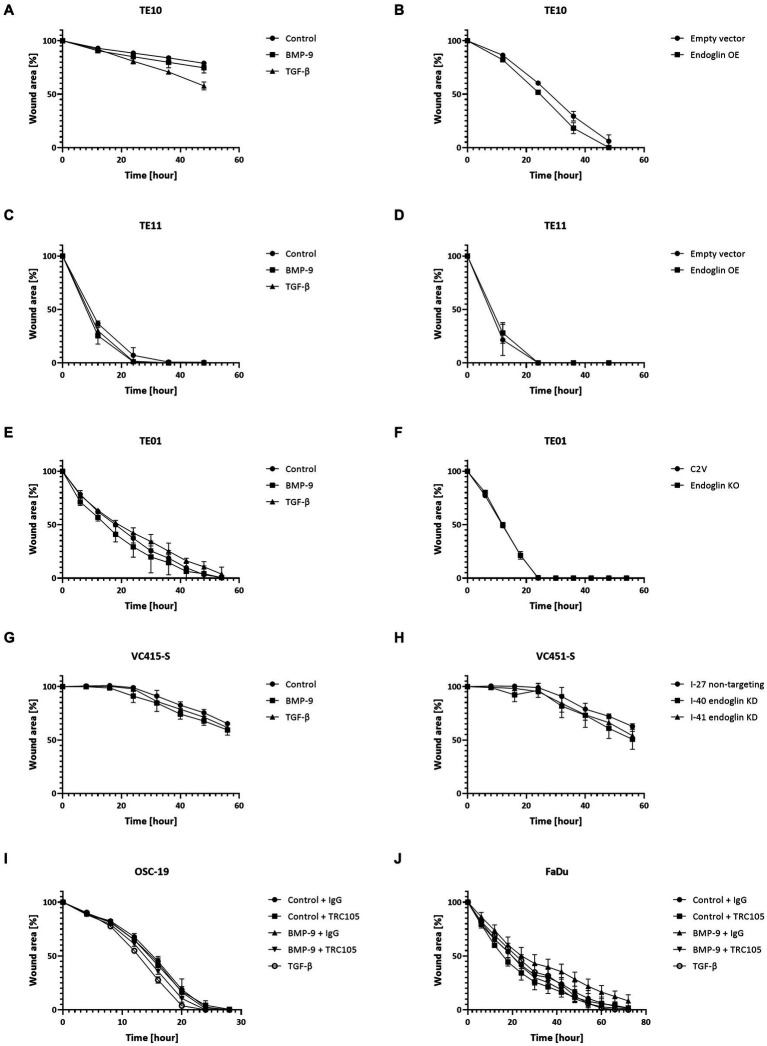
SCC cell lines were stimulated with BMP-9 or TGF-β and the migration of TE10 **(A)**, TE11 **(C)**, TE01 **(E)**, VC415-S **(G)**, OSC-19 **(I)**, and FaDu **(J)** cells was measured via a wound healing assay. To assess the effects of endoglin on cell migration, wound healing assays were performed on endoglin overexpressing (OE) TE10 **(B)** and TE11 **(D)** cells. The effects of endoglin knockout (KO) in TE01 **(F)** and endoglin knockdown (KD) in VC415-S **(H)** were also assessed. Finally, the effects of TRC105 on cell migration was assessed in OSC-19 **(I)** and FaDu cells **(J)**. *n* = 2–3 for each experiment.

Next, we evaluated if endoglin can affect SCC cell migration. Cells were seeded and allowed to reach confluence. Subsequently, a wound was made and the wound closure was followed in time. Given the fact that experiments with mitomycin C, to block proliferation, did not show reproducible data (data not shown) and was accompanied by high toxicity, the effects of proliferation on wound closure cannot be completely excluded. Neither the stimulation with TGF-β or BMP-9 or the OE of endoglin affected cell migration in TE10 and TE11 ESCC cells (Figures 9A–D). Similarly, stimulation with ligands or endoglin KO did not affect cell migration in TE01 (Figures 9E,F). The same was observed for stimulation or endoglin KD in VC415-S (Figures 9G,H). Finally, we evaluated if the endoglin neutralizing antibody TRC105 affects migration of the OSC-19 and FaDu HNSCC cells. BMP-9 did not affect migration, neither did TRC105 affect basal and BMP-9 induced migration (Figures 9I,J). Taken together, these data indicate that endoglin (dependent signaling) does not significantly affect SCC cell line proliferation or migration *in vitro*. A summary of the data, describing the relationship between SCC cell line endoglin expression and BMP-9/TGF-β signaling, cell migration, and cell proliferation, can be found in Table 2.

**Table 2:** Summary table of the relationship between (altered) endoglin expression, BMP-9 signaling, TGF-β signaling, SCC cell migration, and SCC cell proliferation.

Type	Cell line	Endoglin expression	BMP-9 signaling	TGF-β signaling	Cell migration	Cell proliferation
ESCC	TE01	High	ENG KO ↔	ENG KO ↔	ENG KO ↔	ENG KO ↔
ESCC	TE10	Low	ENG OE ↓	ENG OE ↔	ENG OE ↔	ENG OE ↓
ESCC	TE11	Low	ENG OE ↓	ENG OE ↔	ENG OE ↔	ENG OE ↔
VSCC	VC415-C	Low	TRC105 ↓	TRC105 ↔	ND	ND
VSCC	VC415-S	High	TRC105 ↓	TRC105 ↔	ENG KD ↔	ENG KD ↔
HNSCC	OSC-19	Moderate	TRC105 ↔	ND	TRC105 ↔	ND
HNSCC	FaDu	High	TRC105 ↔	ND	TRC105 ↔	ND

ENG, endoglin; KO, knockout; OE, overexpression; TRC015, endoglin neutralizing antibody; KD, knockdown; ND, not determined; ↔ = no effect, ↓ = decrease.

## 4. Discussion

Although the role of endoglin on endothelial cells has been extensively reported, its potential function in SCC has not been determined to the same extent, with only a few reports, often limited to cutaneous SCC (46, 47). Our current data show that endoglin is expressed by epithelial SCC cells in patient samples from HNSCC, ESCC, and VSCC. Interestingly, only individual SCC cells in the tumor express endoglin, while the majority do not. BMP and TGF-β signaling is active in isolated SCC cells, but this seems, except for VSCC cells, independent of endoglin and the type-I receptor ALK1. *In vitro* high expression of endoglin by SCC cell lines leads to endoglin shedding and subsequently inhibition of BMP-9 induced signaling. Finally, endoglin does not seem to influence *in vitro* SCC cell proliferation and migration *in vitro*. However, this does not necessarily mean that endoglin does not affect SCC migration and proliferation *in vivo*.

Interestingly, when we analyzed ESCC, HNSCC, and VSCC patient samples we observed that endoglin expression was restricted to individual cells in the tumor nest. Endoglin has previously been suggested as a cancer stem cell (CSC) marker in renal cell carcinoma (RCC) (48). CSCs are a small population of tumor cells, which are able to self-renew and can reproduce and sustain cancer growth (48). In this previous work, endoglin expressing RCC cells showed increased tumorigenicity in mice when compared to endoglin negative tumor cells. Further analysis of these endoglin positive cells showed several stem cell characteristics (49). These data, together with our findings, might indicate that endoglin could also act as a CSC marker in SCCs as well. Another interesting observation in the SCC patient samples that were analyzed, was increased endoglin expression by VSCC cells with spindle morphology compared to the conventional VSCC cells. Strikingly, it was previously shown in mouse keratinocytes that endoglin shedding induces a spindle cell phenotype (50), while in VSCC, expression seems prominent on the spindle phenotype SCC cells. Importantly, vulvar spindle cell morphology is associated with a worse prognosis than conventional VSCC (10). The selective and high endoglin expression on these spindle shaped cells indicate that endoglin might be a good candidate to identify, image and potentially target these cells in tumors showing spindle cell characteristics.

Squamous cell carcinoma cell lines derived from these SCCs showed variable expression of endoglin. In ESCC, the majority (8/10) of cell lines showed low expression, while in VSCC, exclusively cells with spindle morphology cells show high expression. Surprisingly, none of the SCC cell lines which were analyzed express ALK1. On the contrary, BMP-9 still induced pSMAD1 in most cells. It has however been reported that besides ALK1, BMP-9 can also signal via ALK2 (51–53) and thereby regulate downstream effects. Interestingly, it has been reported that inhibition of ALK2 signaling, effectively suppressed acute myeloid leukemia cell proliferation and migration (54). In contrast it has also been reported that BMP-9/ALK2 signaling can suppress growth of myeloma cells (52). However, in the SCCs that were investigated in the current study, we did not see major effects of BMP-9 on SCC proliferation or migration.

Our data show that BMP-9 signaling is mostly unaffected by endoglin expression, using endoglin KO cells and the endoglin neutralizing antibody TRC105. TRC105 was first introduced in 1999, where treatment with TRC105/drug conjugates resulted in human breast tumor xenograft remission and inhibition of tumor angiogenesis (55) and has undergone clinical development (56). TRC105 works through immune-dependent mechanisms as well as inhibition of endoglin dependent BMP-9 signaling, by competing with BMP-9 binding and inducing endoglin shedding, creating a BMP9 ligand trap (56). These mechanisms contribute to the anti-angiogenic effects of TRC105. Although it does not influence BMP signaling in all SCC cells, TRC105 can selectively target endoglin positive cells and could therefore be used as a tool for targeting SCC cells showing CSC characteristics or imaging of the endoglin high spindle VSCC cells.

Surprisingly we observed that OE of endoglin in low endoglin expressing cells inhibits BMP-9 induced SMAD1 phosphorylation. Endoglin can be shedded from the membrane by MMP-14 (41) and MMP-12 (57) as previously reported, releasing a soluble receptor, which can function as a ligand trap for BMP-9 (52). Soluble endoglin has an anti-angiogenic effect and it has been shown that soluble endoglin levels are upregulated in pre-eclampsia, metabolic disorders, and cancer, although conflicting data have been reported (56, 58–60). Recent work also suggests that when soluble dimeric endoglin binds to BMP-9, it forms a complex that can still actively signal, although, membrane-bound endoglin is required for full signaling to take place (61). Our data suggest that the soluble endoglin, which is generated after OE, binds BMP-9 and acts as a ligand trap, despite high membrane endoglin expression on the cells. Additionally it has also been reported that soluble endoglin is able to inhibit leukocyte adhesion and endothelial transmigration, possibly through binding to integrins (cell surface receptors that play a role in cell–cell/matrix interactions) on the leukocytes (28, 45). Our data using wound healing assays do not suggest a role for soluble endoglin in influencing the migration of SCC cells. Taken together, these show that OE of endoglin leads to increased endoglin shedding and generation of an effective BMP-9 ligand trap.

Although endoglin is expressed by certain SCC cells and more importantly in primary SCC patient samples, endoglin had no effect on *in vitro* cell migration or proliferation of ESCC cell lines. This is in contrast with previous findings that *endoglin (ENG)* was reported to be a tumor-suppressing gene in ESCC. The authors showed that endoglin OE leads to significantly reduced colony formation efficiency, invasion efficiency and tumorigenicity (32). In our work, we saw no to very low endoglin expression on normal esophageal squamous epithelial cells in patient samples. One of the key differences is that we analyzed patient slides, being able to evaluated specific expression of endoglin on SCC cells instead of bulk mRNA, also containing endoglin mRNA from other cells, like the endothelial cells. Furthermore, we did not observe that OE of endoglin affects proliferation or migration of ESCC cells, except for 1 cell line where slightly reduced proliferation upon endoglin OE was observed, which corresponds to the data from Wong et al. They also show high variation in endoglin expression between the cell lines they evaluated and the majority of these data are based on OE of endoglin. Strikingly, endoglin KO or shRNA mediated KD in our hands hardly affects the biological behavior of SCC cells. These somewhat conflicting data, together with the high variation in endoglin expression in the cell lines and selective expression in specific cells in the tumor nests indicate that the role of endoglin remains not completely understood.

In this work, we have focused on autocrine functions of endoglin as a signaling molecule. However, endoglin has also shown to be involved in cell adhesion, by binding to integrins on leukocytes and allowing their extravasation (62), thereby acting in a more paracrine manner. Furthermore, generating high levels of soluble endoglin in the tumor microenvironment might impact the bio-availability of free BMP-9, which can act on the endothelial cells and regulating angiogenesis, cancer-associated fibroblasts and potential regulatory T cells and macrophages (25, 28). Thereby, its expression and subsequent shedding might influence the tumor microenvironment in a paracrine manner.

In conclusion, we found that endoglin is expressed by specific SCC cells in investigated primary SCC tumors and is variably expressed by SCC derived patient cell lines, indicating high heterogeneity. BMP signaling is active in these SCC cell lines, but there seems to be no direct biological effect of altering endoglin expression. Further work should reveal the role of (soluble) endoglin in paracrine signaling in the tumor microenvironment of SCCs.

## Data availability statement

The raw data supporting the conclusions of this article will be made available by the authors, upon reasonable request.

## Ethics statement

Ethical review and approval was not required for the study on human participants in accordance with the local legislation and institutional requirements. Written informed consent for participation was not required for this study in accordance with the national legislation and the institutional requirements.

## Author contributions

SH, SJ, MT, MG, EJ-M, SC, TH, JB, WD, MS, and LH made substantial contributions to conception and design, acquisition of data, or analysis and interpretation of data, took part in drafting the article or revising it critically for important intellectual content, agreed to submit to the current journal, gave final approval of the version to be published, and agree to be accountable for all aspects of the work.

## Funding

SH was supported by a research grant from the Flanders Agency for Research and Innovation (VLAIO-HBC.2018.2002).

## Conflict of interest

SH was employed by InnoSer België NV, outside the submitted work. LH reports sponsored research grants from TRACON Pharmaceuticals, not related to the work in this study. In addition, LH is coinventor on a patent on the combination of TRC105 with PD1 therapy issued to TRACON.

The remaining authors declare that the research was conducted in the absence of any commercial or financial relationships that could be construed as a potential conflict of interest.

## Publisher’s note

All claims expressed in this article are solely those of the authors and do not necessarily represent those of their affiliated organizations, or those of the publisher, the editors and the reviewers. Any product that may be evaluated in this article, or claim that may be made by its manufacturer, is not guaranteed or endorsed by the publisher.

## Abbreviations

ALK: Activin receptor-like kinase
BMPs: Bone morphogenic proteins
BSA: Bovine serum albumin
BSL-2: Biosafety level 2 laboratory
CAFs: Cancer-associated fibroblasts
CSC: Cancer stem cell
cSCCs: Cutaneous squamous cell carcinomas
ESCCs: Esophageal squamous cell carcinomas
FFPE: Formalin fixed paraffin-embedded
HNSCCs: Head and neck squamous cell carcinomas
HPV: Human papillomavirus
KD: Knockdown
KO: Knockout
LUMC: Leiden University Medical Center
OE: Overexpression
PBS: Phosphate-buffered saline
qPCR: Real-time quantitative PCR
RCC: Renal cell carcinoma
RPMI: Roswell Park Memorial Institute
SCC: Squamous cell carcinoma
shRNA: Short hairpin RNAs
TGF-β: Transforming growth factor beta
VSCCs: Vulvar squamous cell carcinomas
