# Supplementary material for: Endoglin and squamous cell carcinomas
Source: Front Med (Lausanne). 2023 Jun 16;10:1112573. doi: 10.3389/fmed.2023.1112573 (PMC10313935; doi:10.3389/fmed.2023.1112573)
Supplement: Supplementary file 1 [file Table_1.DOCX]

Supplementary Table 1. Primer sequences.

| **Gene** | **Forward primer (5’ --- 3’)** | **Reverse primer (5’ --- 3’)** |
| --- | --- | --- |
| *ACTB* | GCACAGAGCCTCGCCTT | GTTGTCGACGACGAGCG |
| *ENG* | CCGAGAGGTGCTTCTGGTCC | GTGCAGTGGGATTCCCAGG |
| *ALK1* | CTGGTTCCGGGAGACTGAGAT | TGCGGGAGGTCATGTCTGA |
| *ALK2* | GACGTGGAGTATGGCACTATCG | CACTCCAACAGTGTAATCTGGCG |
| *ALK3* | GGGGTCCGACTTATGAAA | TACGACTCCTCCAAGATGTGG |
| *ALK4* | CACCTCAGGGTCTGGCTC | AACCAAGACCGTTCTTCACG |
| *ALK5* | ACGGCGTTACAGTGTTTCTG | GCACATACAAACGGCCTATCT |
| *ALK6* | GCTCAGGAAGTGGATCAGG | CATGCCTCATCAACACTGTC |
| *ALK7* | CGCACTTCAAAAGGGTGTCG | TGATGCCCAACATGCTCCTT |
