# Supplementary material for: Endoglin and squamous cell carcinomas
Source: Front Med (Lausanne). 2023 Jun 16;10:1112573. doi: 10.3389/fmed.2023.1112573 (PMC10313935; doi:10.3389/fmed.2023.1112573)
Supplement: Supplementary file 2 [file Data_Sheet_1.DOCX]

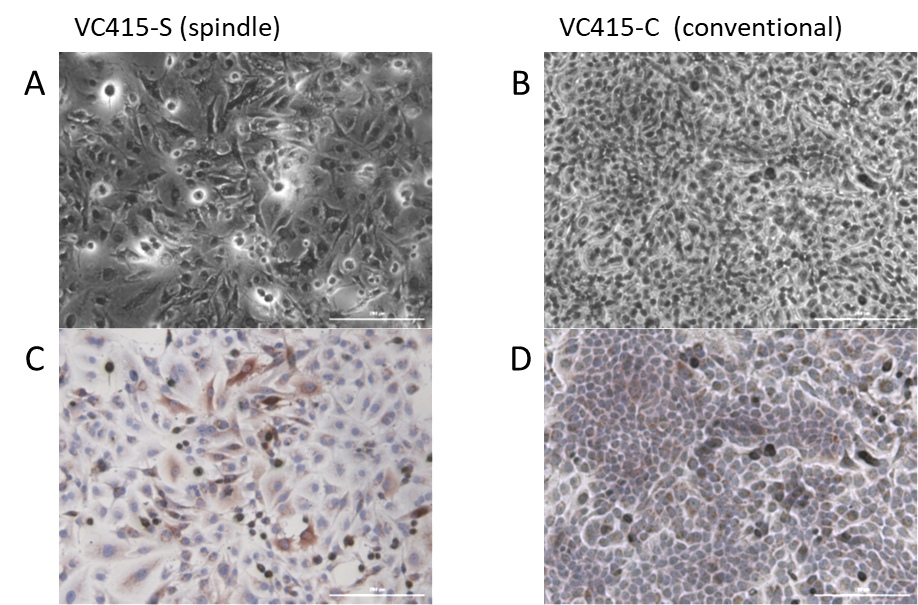


Supplementary Figure 1. Images of the morphologies of VSCC spindle and conventional cells. Phase contrast images of spindle (**A**) and conventional (**B**) VSCC cells, isolated from the same patient tumor. Cells were stained for pan-cytokeratin expression via IHC (**C-D**). All images taken at 100x magnification.
