# Supplementary material for: Endoglin and squamous cell carcinomas
Source: Front Med (Lausanne). 2023 Jun 16;10:1112573. doi: 10.3389/fmed.2023.1112573 (PMC10313935; doi:10.3389/fmed.2023.1112573)
Supplement: Supplementary file 3 [file Data_Sheet_2.DOCX]

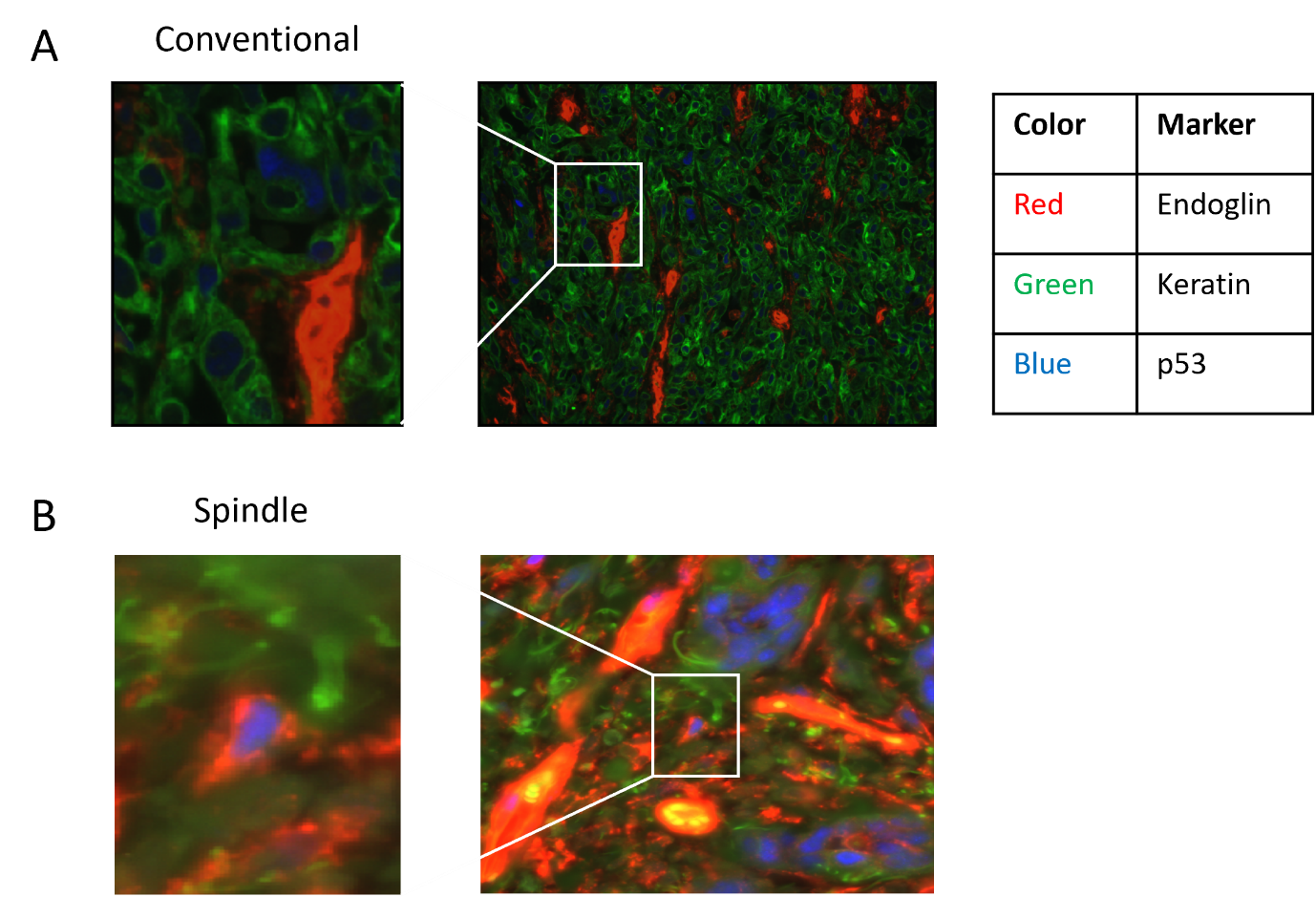


Supplementary Figure 2. Triple immunofluorescent staining for endoglin, keratin and p53 on vulvar cancer tissue. Panel (**A**) shows intense staining of endoglin in endothelial cells (no p53 staining) and no staining in epithelial conventional tumor cells, which stain green. In panel (**B**), clear co-localisation of endoglin and ‘mutant’ p53 in the same spindle cell, indicating epithelial origin of the endoglin expressing spindle-like tumor cells.
