# Supplementary material for: Endoglin and squamous cell carcinomas
Source: Front Med (Lausanne). 2023 Jun 16;10:1112573. doi: 10.3389/fmed.2023.1112573 (PMC10313935; doi:10.3389/fmed.2023.1112573)
Supplement: Supplementary file 4 [file Data_Sheet_3.DOCX]

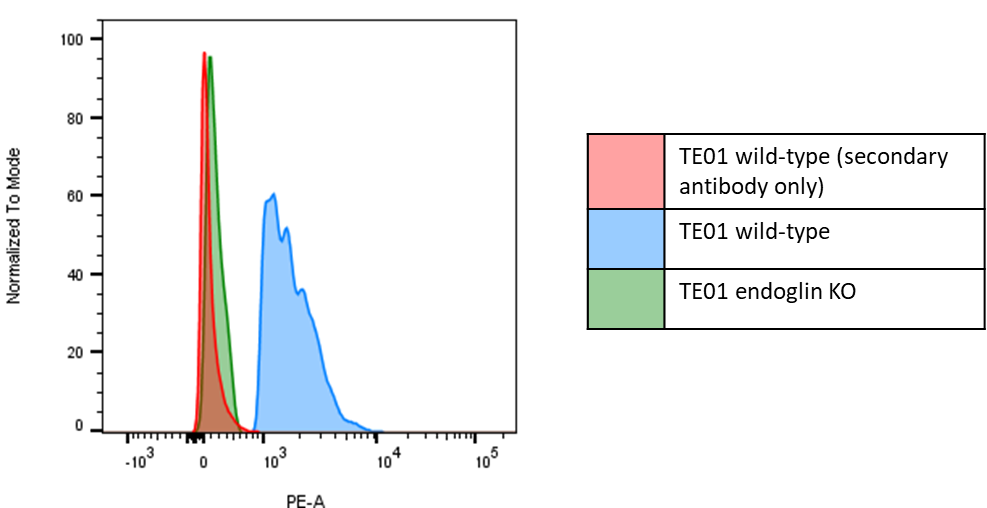


Supplementary Figure 3. FACS histogram of the verification of endoglin knock-out (KO) in ESCC cell line TE01. Endoglin expression by TE01 endoglin KO cells, which was compared to wild-type TE01 cells and wild-type TE01 cells that were only exposed to the secondary antibody.
