# Supplementary material for: Endoglin and squamous cell carcinomas
Source: Front Med (Lausanne). 2023 Jun 16;10:1112573. doi: 10.3389/fmed.2023.1112573 (PMC10313935; doi:10.3389/fmed.2023.1112573)
Supplement: Supplementary file 5 [file Data_Sheet_4.DOCX]

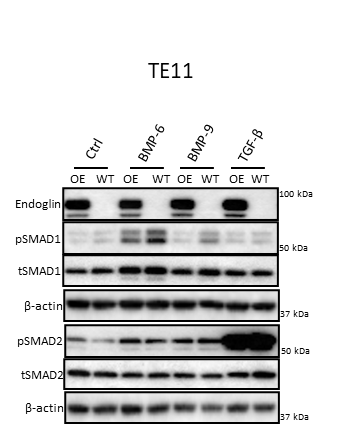


Supplementary Figure 4. TE11 with and without endoglin overexpression (OE) cells were stimulated with either BMP-6, BMP-9 or TGF-β. pSMAD = phosphorylated SMAD, tSMAD = total SMAD.
